# Ultrasonographically diagnosed diaphragmatic dysfunction and weaning failure from mechanical ventilation in critically ill patients

**DOI:** 10.1186/2197-425X-3-S1-A454

**Published:** 2015-10-01

**Authors:** B-P Dubé, A Demoule, J Mayaux, D Reuter, H Prodanovic, T Similowski, M Dres

**Affiliations:** 1 Sorbonne Universités, UPMC Université Paris 06, INSERM, UMRS 1158 Neurophysiologie Respiratoire Expérimentale et Clinique, Paris, France; 2 AP-HP, Groupe Hospitalier Pitié-Salpêtrière Charles Foix, Service de Pneumologie et Réanimation Médicale (Département 'R3S'), Paris, France

## Introduction

Clinical data suggest that diaphragmatic dysfunction (DD) is associated with difficult weaning from mechanical ventilation. However, studies focusing specifically on diaphragmatic function in this setting are scarce.

OBJECTIVE

To predict the outcome of a spontaneous breathing trial (SBT) through the ultrasonographic assessment of diaphragmatic and intercostal muscle function.

## Methods

Mechanically ventilated patients from a 16-bed medical intensive care unit (ICU) were prospectively included before starting an SBT. Diaphragmatic function was estimated by the intrathoracic depression induced by anterior magnetic phrenic stimulation (Ptr,stim) and by performing an ultrasonography of the right hemidiaphragm. Intercostal muscle function was assessed using ultrasonography of the second right anterior intercostal space. We measured expiratory and peak inspiratory muscle thickness (T_de_ and T_di_ respectively) and muscle thickening fraction (TF_D_ and TF_IC_ for diaphragm and intercostal), defined as (T_di_ - T_de_)/T_di_.

The Medical Research Council (MRC) Score was also used to detect peripheral muscle weakness.

Successful weaning (SW) was defined by extubation after SBT without reintubation in the following 48 hours and weaning failure (WF) by a failed SBT or a reintubation in the 48 hours after extubation. DD was defined by a value of Ptr,stim of less than -11 cmH_2_O.

## Results

Forty patients were included (age 55 ± 18): 27 and 13 patients in the SW and WF group respectively. Mean SOFA score was 5 ± 3 and length of mechanical ventilation the day of the SBT was 7 ± 3 days.

Compared to SW patients, WF patients had lower Ptr,stim (6.1 ± 0.7 vs 13.6 ± 1.2 cmH_2_O p < 0.001), lower TF_D_ (21.2 ± 6 vs 35.6 ± 13% p < 0.01), lower MRC score (40.3 vs 55.7, p < 0.01) and higher TF_IC_ (26.7 ± 15 vs 10.4 ± 6%, p < 0.01). Areas under the receiver operating characteristics curves to predict WF were 0.86, 0.84, 0.88 and 0.88 for Ptr,stim, TF_D_, MRC score and TF_IC_ respectively (all p < 0.05). The best Ptr,stim and TF_D_ thresholds to predict WF were 8.2 cm H_2_O and 29%, respectively.

Ptr,stim was significantly correlated with TFd, MRC score and TFic (rho = 0.88, 0.55 and -0.90 respectively).

Among patients with DD, only 46% were successfully separated from the ventilator whereas all patients without DD were successfully separated from the ventilator (p < 0.001).

## Conclusions

Ptr,stim, TF_D_, MRC and TF_IC_ are strong predictors of weaning outcome.

Our findings support the hypothesis that diaphragmatic dysfunction is significantly, although not systematically, associated with weaning failure.

## Grant Acknowledgment

Martin DRES was supported by Assistance Publique Hôpitaux de Paris

